# Understanding Mental Health Apps for Youth: Focus Group Study With Latinx Youth

**DOI:** 10.2196/40726

**Published:** 2022-10-18

**Authors:** Elena Agapie, Katherine Chang, Sneha Patrachari, Martha Neary, Stephen M Schueller

**Affiliations:** 1 Department of Informatics University of California, Irvine Irvine, CA United States; 2 Department of Psychological Science University of California, Irvine Irvine, CA United States

**Keywords:** mental health, mental health apps, youth, child, teenager, focus group, human-centered design, mobile health, mHealth, health app, cognitive behavioral therapy, CBT, perspective, qualitative, mindfulness, health app, digital health tool, Latino, Latinx, mobile phone

## Abstract

**Background:**

An increasing number of mental health apps (MHapps) are being developed for youth. In addition, youth are high users of both technologies and MHapps. However, little is known about their perspectives on MHapps. MHapps might be particularly well suited to reach the youth underserved by traditional mental health resources, and incorporating their perspectives is especially critical to ensure such tools are useful to them.

**Objective:**

The goal of this study was to develop and pilot a process for eliciting youth perspectives on MHapps in a structured and collaborative way. We also sought to generate learnings on the perspectives of Latinx youth on MHapps and their use in ways that might facilitate discovery, activation, or engagement in MHapps, especially in Latinx populations.

**Methods:**

We created a series of focus groups consisting of 5 sessions. The groups introduced different categories of MHapps (cognitive behavioral therapy apps, mindfulness apps, and miscellaneous apps). Within each category, we selected 4 MHapps that participants chose to use for a week and provided feedback through both between-session and in-session activities. We recruited 5 youths ranging in age from 15 to 21 (mean 18, SD 2.2) years. All the participants identified as Hispanic or Latinx. After completing all 5 focus groups, the participants completed a brief questionnaire to gather their impressions of the apps they had used.

**Results:**

Our focus group methodology collected detailed and diverse information about youth perspectives on MHapps. However, we did identify some aspects of our methods that were less successful at engaging the youth, such as our between-session activities. The Latinx youth in our study wanted apps that were accessible, relatable, youth centric, and simple and could be integrated with their offline lives. We also found that the mindfulness apps were viewed most favorably but that the miscellaneous and cognitive behavioral therapy apps were viewed as more impactful.

**Conclusions:**

Eliciting youth feedback on MHapps is critical if these apps are going to serve a role in supporting their mental health and well-being. We refined a process for collecting feedback from the youth and identified factors that were important to a set of Latinx youth. Future work could be broader, that is, recruit larger samples of more diverse youth, or deeper, that is, collect more information from each youth around interests, needs, barriers, or facilitators or better understand the various impacts of MHapps by using qualitative and quantitative measures. Nevertheless, this study advances the formative understanding of how the youth, particularly Latinx youth, might be viewing these tools.

## Introduction

### Background

Numerous health apps exist, with estimates of just over 350,000 health apps in 2020, with 18% of those being for stress and wellness and 5% for mental health conditions [1]. Despite this large number of available mental health apps (MHapps), few are downloaded, and those that are downloaded rarely have long-term, sustained use. A review of MHapps targeting depression and anxiety found that the top 3 most downloaded apps accounted for 90% of all downloads and that 63% of depression apps and 56% of anxiety apps had no regular users over a month period [2]. Another study found that the median retention rate for MHapps 15 days after downloading was 3.9%, and by 30 days, it dropped to 3.3% [3]. Thus, app discovery (ie, finding useful MHapps), app activation (ie, initiating the use of an MHapp), and app engagement (ie, sustained and effective use of an MHapp over time) are all major barriers to ensuring that MHapps can provide the most impact on consumers’ lives.

It is often proposed that MHapps could have a significant impact on youth [4-6]. Most mental health disorders emerge in adolescence or early adulthood, suggesting that youth is a critical transitory period for mental health [7]. Youth face many barriers to care that include structural and perceptual barriers, including perceptions of both mental health problems and services [8]. Youth of color experience even more barriers to care. They are less likely to live in communities with mental health providers [9] and to receive traditional mental health care than their White counterparts [10,11]. Even when providers and treatments are available, providers are less likely to match their racial and ethnic backgrounds, and culturally relevant care is rarely available [12,13]. For youth with mental health needs, parents and other adults are often the gatekeepers to care. As such, youth often seek care through informal sources first, which includes family and friends but increasingly includes internet-based information and interventions [14]. Seeking informal sources of support before engaging in traditional care might be even more common among Black and Latinx youth than among their White counterparts [15].

MHapps are a potentially useful way to reach youth and provide mental health support and interventions. Smartphone ownership increases substantially from age 11 to 18 years. With over half of youth aged 11 years and 91% of those aged 18 years owning smartphones [16]. As such, youth are quite comfortable using technology, and most report using them almost constantly to access the internet and communicate with peers [17]. Many youth also report that they are using or have used health apps. A recent representative survey of those aged 14 to 22 years found that 69% report having used a health app, a number that climbs to 75% among the youth with moderate to severe symptoms of depression [18]. In this survey, many of the categories of apps youth reported using were related to mental health, including sleep (27%), meditation or mindfulness (17%), stress reduction (14%), mood tracking (10%), depression (9%), and alcohol or substance use (5%). Thus, MHapp use among youth seems common.

MHapps might be particularly useful to provide mental health resources for traditionally underserved groups, such as racial and ethnic minorities [19,20]. First, as mentioned earlier, a sufficient workforce that mirrors the racial and ethnic diversity of the population in need does not exist. As an example, in California, Latinxs represent 38% of the state’s population but only 4% of its psychiatrists and 8% of its psychologists [21]. Investment in developing a better pipeline to train and retain diverse mental health providers should be a priority, although this process will take time and resources are needed today to help those in need. Second, MHapps can overcome geographic barriers and can be deployed more effectively where gaps in service provision exist. More than 80% of counties in the United States are designated as mental health professional shortage areas [22], and again, youth of color are less likely to live in areas with access to care [9]. Third, technology might be seen as a more desirable resource either because of the ease of access, overcoming barriers such as transportation or stigma [20], or just because some people might be more inclined to use an MHapp rather than traditional care. Calls to use technology for mental health service delivery have emphasized the need to broaden the portfolio of available mental health resources to promote market segmentation; that is, identifying targeted groups of consumers to better tailor products, such as mental health resources, to those populations. Even when racial and ethnic minorities receive traditional mental health services, they tend to receive fewer sessions [23]. Therefore, in addition to considering how to better engage racial and ethnic minorities in care, we must also consider how to design care that will better serve them when they get there. MHapps might be one such solution to help address this challenge.

Although robust literature exists to suggest that MHapps are effective [24,25], reviews specifically focused on apps for youth have shown that research in this space is sparse [26,27] but have demonstrated some indications of positive benefits [28]. In addition to there being fewer studies that address the effectiveness of MHapps for youth, most of the studies conducted focus on the early feasibility or acceptability of apps that never make it into the hands of consumers. For example, Grist et al [27] found in their systematic review that of the 15 apps described in 24 eligible studies, only 2 were available to download. Studies on MHapps for youth have been characterized by poor uptake and engagement, lack of specification of procedures such as human support, and a lack of research on younger children or traditionally marginalized populations [29]. Given the lack of empirical evidence to speak of the effectiveness of MHapps for racial and ethnic minority youth, processes to better elicit their perspectives and views could be useful to inform the adoption and deployment of MHapps among such populations.

Thus, a gap exists between the use and enthusiasm of MHapps for youth and our understanding of the actual impact on this group. It is worthwhile to better understand youth’s impressions, especially youth of color, of MHapps to understand what they would use and why, potentially leading to better design and dissemination of MHapps for youth and youth of color.

### Including Youth Perspectives in the Research Process

One way to address this gap and provide information to better design studies to investigate the potential effectiveness of MHapps for youth is to incorporate youth’s perspectives on MHapps, especially those currently available to youth, into research [30]. Indeed, research has taken different approaches to consider the youth perspective, including expert opinions, app reviews, and working with youth themselves. One study conducted a workshop with psychologists with experience working with youth to identify different youth media preferences and then analyzed app features to identify the presence of these media preferences [31]. Media preferences included strategies of social connectivity, use of videos, tailorability, and rich interactions. However, they found that the use of these strategies was limited in apps available for youth, with social connectivity being the most used but with few examples of interactive multimedia experiences. Several studies have worked with youth to co-design digital mental health interventions [32-35], including MHapps, but these studies have often resulted in early-stage ideas or functional prototypes that, although sometimes are evaluated, rarely make it to app stores or other places where youth could access them. Finally, many studies have focused on trying to understand MHapps from app store user reviews [36,37]. Although these studies have not focused specifically on MHapps for youth people or on reviews coming only from youth, given that youth are using these tools, it is possible that some of these reviews come from youth. Overall, these studies demonstrate findings similar to other evaluations that leverage expert opinions or content analysis. Users appreciate apps that are esthetically pleasing and functionally diverse while allowing opportunities for personalization and tailoring. In addition, users dislike apps with poor usability and pricing models that focus on freemium models or emergent costs. However, a limitation of these reviews is that these prompts are general and may not elicit feedback on the aspects of MHapps that might best support their design and implementation.

Diverse methods exist to gather people’s impressions of digital products and to team with potential users in ways to better create technologies that meet their needs. These methods, leveraged from the field of human-centered design, have been used in various projects working to develop MHapps for the youth or to understand the needs and interests of the youth when interacting with technologies. Applying such techniques to better understand the impressions of existing apps is also useful to understand what are the affordances of these technologies as well as where such technologies are lacking. In our study, we drew on synchronous methods, used focus groups, and adapted asynchronous remote community (ARC) methods to inform the aspects of our study design. ARC includes a collection of methods intended to facilitate group-based research at a given distance [38]. ARC has been used in various apps, especially for working with populations in which in-person coordination might be challenging, such as populations with rare diseases [39] or stigmatized populations [40]. ARC methods have also been used with teens to leverage digital communication using platforms familiar to teens (eg, social media and asynchronous communication) and to balance their busy schedules [41]. Despite the creation of ARC methods before the pandemic, these methods are also useful for conducting remote design work that is necessary, given the need to follow physical distancing protocols.

In this study, we attempted to develop a process for incorporating youth perspectives on MHapps in a structured and collaborative way. We sought to pilot our methods with a small group of youth while also attempting to learn their perspectives on available MHapps. We were particularly interested in the views of youth from traditionally underserved and marginalized populations (given the potential of MHapps to overcome barriers they face and the relative lack of knowledge of MHapps for these populations in comparison with their White counterparts) and therefore worked with a sample of youth with a Latinx background. We conducted a 5-week focus group with a set of youth aged 15 to 21 years to refine a process for incorporating app feedback into their views on MHapps and to identify some characteristics of MHapps that might serve as barriers to or facilitators of discovery, activation, or engagement. As such, we framed our work in contributing both our methodologies and some formative learnings from our small pilot of these methods.

## Methods

### Recruitment

We recruited participants through a local nonprofit team that works with youth from underrepresented groups, especially Latinx youth, to accelerate readiness in Science, Technology, Engineering, Arts, and Math. Recruitment occurred via email and recruitment messages posted on the nonprofit team’s Discord channel. We compensated participants with a US $25 Amazon gift card for each of the 5 focus group sessions that a participant attended, with a maximum compensation of US $125 for the entire study.

### Ethical Considerations

Informed consent was obtained from all participants aged ≥18 years. For participants <18 years of age, informed consent was obtained from their parent or guardian and assent was obtained from the participants. Researchers had an existing partnership with this nonprofit team, which facilitated consenting participants and their parents or guardians for participants <18 years. A member of our research team provided a digital consent or assent form to the participants and a digital consent form to parents for participants <18 years of age. A member of the nonprofit team helped facilitate contact with parents or guardians when necessary. Institutional review board approval was obtained from the University of California, Irvine (#2019-5609).

### In-Session Activities

We conducted 5 focus group sessions, 1 per week, totaling 5 weeks. For the first 3 weeks, each week, participants used a new category of MHapps and then discussed the app they used during the following session. In the fourth week, participants were asked to choose 1 app to use from all the apps we introduced across all the weeks. In each session, we collected feedback about the apps participants used in the past week. In the fifth session, we discussed feedback across all the apps participants used.

The app categories were cognitive behavioral therapy (CBT) apps (week 1), mindfulness apps (week 2), and miscellaneous apps, which did not pertain to 1 category, including a coping app, journaling app, mood-tracking app, and peer support app (week 3). Each category contained 4 apps. These apps were selected to represent the general categories of MHapps that the youth use and the features present in popular MHapps. Furthermore, our research team used our experience of identifying and evaluating apps and considered apps that were used by youth based on previous analyses [42]. Each of the 4 apps discussed in the week was assigned to a different participant (2 participants used the same app).

We selected focus groups for our method because focus groups enabled us to contrast between the different apps by engaging in group discussions. We used this approach to enable discussions about the different types of functionalities of related apps. Focus groups also enabled us to address known challenges in working with teens [43] by making it easier to balance power dynamics between researchers and teens. We drew on design theory that has established that discussion around different prototypes can result in increased participant rapport and sharing about the prototypes they each engaged with [44]. We also built on prior work through which exposing participants to different types of designs and functionalities can elicit feedback on functionalities that are most important to users [45].

### Focus Group Session Structure

Each focus group session was approximately 90 minutes and was organized around a similar schedule (Table 1): icebreakers to build rapport between participants and researchers, discussion and activities about the apps assigned to participants in the past week (week 2-5), a brief presentation of a core concept for the apps in the upcoming week (eg, CBT or mindfulness), and assignment of apps that participants would use in the following week. To ensure that all the apps were evaluated, each participant chose a different app until all the apps were chosen by at least one person. We then asked the participants to use the app of their choice throughout the next week. In the week 4 session, the participants could choose an app that they had already used in the past few weeks or they could choose a completely new app that they were interested in.

**Table 1:** Overview of session topics and apps assigned.

Week	Activities in focus group sessions	MHapps^a^ assigned
Week 1	Overview of the study Getting to know each other (icebreaker) Introduction to CBT^b^	Mindshift Sanvello Woebot Wysa
Week 2	Getting to know each other (icebreaker) Feedback on CBT apps Introduction to mindfulness	Headspace Insight Timer Shine Smiling Mind
Week 3	Getting to know each other (icebreaker) Feedback on mindfulness apps Introduction to “wellness hacks”	Covid Coach Daylio Moodflow Talk Life
Week 4	Getting to know each other (icebreaker) Feedback on miscellaneous apps Overview of last week’s assignments Review and selection of previous weeks’ apps	An app from a previous week
Week 5	Getting to know each other (icebreaker) Reflecting on all apps App design activity	N/A^c^

^a^MHapp: mental health app.

^b^CBT: cognitive behavioral therapy.

^c^N/A: not applicable.

### Focus Group Activities for Collecting Feedback

We collected feedback about participants’ experiences using the assigned apps in the past week for approximately 50 of the total 90 minutes of sessions in weeks 2 to 5. Participants filled out sticky notes on an interactive remote whiteboard named Miro [46] in response to guided questions. Participants then shared their experiences with the app they used orally with the rest of the group. We used the following questions to guide the discussion: “What did you like most about the app?”, “What did you like least?”, “How do you think apps like this could be useful for youth like you?”, “What do you think apps like this are missing for youth like you?”, and “If you could design a [category, i.e., CBT, mindfulness, mental health]-app for youth like you, what would you do differently?”

### Between-Session Activities

**Figure 1:**
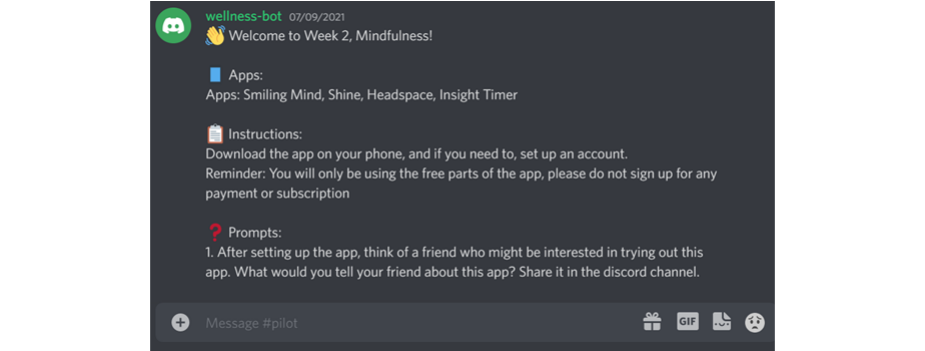
Example message sent to participants through the Discord platform.

We used a web-based community platform to communicate with participants and elicit further information from participants about their experiences using the app they chose to interact with for the week. We chose Discord [47] because the participants were already using it as part of the community from which we recruited. The goal of these activities was to capture participant perspectives as they were using the apps in their lives, rather than reflecting on their use in the focus group sessions. We planned to deliver 3 questions every week. We used a generic user profile called “wellness-bot,” which would post the questions in Discord (Figure 1). The profile was controlled by a research team member. We named the account wellness-bot to encourage users to interact with the account as if it was a chatbot and feel more comfortable with the anonymity of sharing with a chatbot instead of a person. Some questions were delivered in a channel created for this study that all participants shared, while others were delivered through direct messages. The questions prompted the participants to use the app and provide feedback on their experiences using the app.

Engagement in Discord started immediately after the first focus group. We continued that week with the second question:

Now that you’ve set up the app, try out an activity! Some examples include logging how you’re feeling today, reviewing past entries, listening to a video, etc. What is something about the app that you find useful or not useful? Share in the channel.

### Follow-up Survey

At the end of participation, the participants received a follow-up survey about the apps they used. They were asked to indicate the app they used within each category. For each app they indicated, they were asked the following questions (response options) shown in Textbox 1.

This survey was created by adapting the One Mind PsyberGuide Consumer Review Questionnaire [48] and questions from the Mobile App Rating Scale [49], which have been recommended by service users for consumer evaluation in another study [48]. This included star ratings, satisfaction as indicated by likelihood to recommend the product, and open-ended questions about what they liked the most and least about the app. These questions also align with those used to evaluate technologies and apps in other settings, such as star ratings from app stores or the Net Promoter Score.

**Follow-up survey about the apps they used.**

1. During the week you used the app, how often did you use it? (1, One day of the week; 2, A few days of the week; 3, Once a day; 4, Multiples times per day)
2. What did you like most about the app? (Free-text response)
3. What did you like least about the app? (Free-text response)
4. What impact, if any, did this app have on your wellness or how you felt? (1, Negative impact; 2, No impact; 3, Small positive impact; 4, Moderate to large positive impact)
5. Did you continue to use this app after the week you were asked to use it? (1-No; 2-Yes)
6. Would you recommend this app to people who might benefit from it? (1, Not at all--I would not recommend this app to anyone; 2, There are very few people I would recommend this app to; 3, Maybe -- There are several people whom I would recommend it to; 4, There are many people I would recommend this app to; 5, Definitely -- I would recommend this app to everyone)
7. What is your overall star rating of the app? (1, ☆ -- One of the worst apps I’ve used; 2, ☆☆; 3, ☆☆☆ -- Average; 4, ☆☆☆☆; 5, ☆☆☆☆☆ -- One of the best apps I’ve used)

### Analytic Strategy

#### Qualitative Analysis of Feedback From the Youth

We used thematic analysis to analyze the focus group data, using an inductive approach [50] to conceptualize themes. A senior (EA) and junior (SP) researcher open coded the focus group transcripts using a descriptive coding approach [51]. The codes described the topics mentioned by the participants on a line-by-line basis. The codes were identified inductively based on the data. Given the small sample of data, we did not follow a fixed codebook; rather, we coded for the different topics that participants discussed and then conceptualized themes. Examples of codes identified include easy-to-consume content, personalization, social interaction, and relatability of content. The 2 researchers worked closely together, discussing data from all the focus groups. On the basis of the resultant codes, the 2 researchers conceptualized the data into themes related to each other through brief memos summarizing the coded data. During weekly meetings, the researchers discussed codes and themes in the data. The 2 researchers grouped subthemes together into themes such as personalization, connecting with others, and reliability of content. The other 4 researchers in the team assessed the subthemes and provided feedback, which led to a slight reorganization of the themes as presented in the results. Because the data collected centered on feedback about the functionalities of the apps, the themes that were conceptualized are also connected to app functionalities (eg, content type, features, and design of the app). We did not conduct interrater reliability, as any misalignments in coding supported our process of synthesizing data, understanding themes, and revising memos [52]. The themes presented are comprehensive of our sample, but given the small sample size and formative nature of this research, we did not intend to reach data saturation.

#### Analysis of Research Approach

The research team reflected on the stages of the research design and assessed which ones were successful at engaging the youth: the way we engaged and built rapport with youth, how we engaged with the group of participants, and the activities we designed during and between the focus group sessions. Because this was a preliminary and formative study, our research team met on a weekly basis to discuss that week’s session, make any modifications to the next week’s activities, and discuss lessons learned. We note that our team conducting the research procedures did not include any members that identified as Latinx. However, this study was part of an existing research partnership with a nonprofit team from which participants were recruited, and members of the nonprofit team shaped the research design and recruitment considerations. For example, together we agreed that focus groups were suitable and to use the nonprofit team’s existing Discord infrastructure for between-session interaction. The nonprofit team’s members also directed our recruitment to students who were part of the program, influencing who was willing to participate. The research team included 1 researcher who had been conducting qualitative studies for >10 years (EA) and another for >5 years (SMS). In addition to collaboration with this nonprofit team for this project, our team also had multiple years of research and clinical training with Latinx communities and collaboration with Latinx investigators.

## Results

### Participants

Five participants attended each of the focus groups. The participants ranged in age from 15 to 21 (mean 18, SD 2.2) years. Of 5 participants, 4 (80%) identified as women and 1 (20%) as men. All the participants identified as Hispanic or Latinx, and 1 (20%) participant also identified as Black.

### Focus Group Insights

#### Overview

Participants desired a range of features that would tailor the apps to the needs and practices of the youth, such as topically relevant content to youth issues, lightweight and playful content, and brief interactive exercises or content. The youth noted that the content of the apps came from authoritative sources. The youth also emphasized the importance of authentic social connections with others.

#### Personalizing Content With Youth’s Needs

Participants shared that they often found the content of these apps not specific enough to the problems that youth encountered. Users mentioned wanting apps specifically designed for their demographics, age, challenges, and stage of life. One of the participants currently enrolled in high school felt “different timelines or milestones that youth deal with, [specifically] graduating from high school, and navigating adult life” could be helpful for others in the youth community.

#### Customizing Content and App Display

The participants found that tailoring the amount of content to their particular interests would help them engage with the apps. Such useful tailoring occurred when participants could customize the topics shown in the app so that they were better aligned with their interests (P4). The participants also wanted to customize the look and feel of the app by changing the colors used in the app (P4 and P1). They also preferred topics customized to the user, for example, by avoiding sensitive topics:

I took into consideration that some people might have trauma or something. And it was like, oh, would you rather not see posts related to certain topics. So they pretty much asked you that, before you even get to see anything, which I really liked.

P4

The participants valued activities that could capture their personal situations. For example, P4 valued having the opportunity to create a plan to manage anxiety that was personal to themselves, not just to listen to or read generic content about the topic. When the content fit the participant’s life, they felt the app was more relatable:

I clicked on one of the manage stress [short audio clips] and they all let me see, oh, that’s my story too.

P1

#### Content Aligned to Youth Use of Technology: “Lighten the Mood”

The participants hoped that MHapps would complement the types of activities they engaged in when using technology. They wanted the duration of the content to be aligned with their use of technology for entertainment:

Nowadays, people want something entertaining over something that takes more time. So in order for...youth to want to use a wellness app, they would have to bring in entertainment...maybe...including games, like something to make it more fun.

P5

Another participant mentioned that it was important to have content to “lighten the mood” and make it “light on emotion,” such as comedic content, or visual and interactive elements, such as emojis and stickers (P2). They thought this could be more aligned with a positive interaction with technology:

I feel like this type of app should feel more like, I want to do this [as] part of my daily routine that’s actually going to bring me joy.

P2

They thought that content related to mental health could be alternated with other types of engaging content:

Maybe I don’t want to be asked how I’m doing. Maybe I just want to play a game.

P1

A different way of making content more lightweight was through encouraging messages:

As soon as I woke up the notification with words of encouragement was already there. And I would just have to click on it, and it would take me to a meditation.

P5

### Integration With Offline Activities

#### Overview

Several participants mentioned that they did not carry their phones with them all the time or that they preferred to do certain activities supported by the apps in a nondigital context; for example, journaling on paper. Some participants (P1 and P2) did not foresee using the digital tool for journaling:

I don’t really use my phone for any other apps, mainly just internet browsing and YouTube.

P1

One participant thought it could be useful to get complementary support in their journaling activities; for example, by getting journaling templates that can guide the activity (P2).

#### Valuing Diversity in Content Over Single-Focus Apps

Participants found it valuable to have access to a range of content because it could give them control over what features to use:

There was a lot of different categories, and I just chose the ones that I was, more like I needed help with.

P4

The type of content that interested participants included informational articles, audio content, meditation videos, or social content to help them relate to other people’s experiences (P4 and P5). The participants valued seeing content on different topics, such as work, exercise, sleep, nutrition, mindfulness, emotions, or managing the pandemic (P3, P4, and P5) and expressed reluctance in using an app that was focused on only 1 topic, such as anxiety (P3 and P4). They preferred entertaining features in apps. They wanted access to music playlists to boost their mood (P4).

#### Connecting With Others: Valuing Receiving and Providing Authentic Support

The participants valued apps that had a social component, making them feel less alone (P2, P4, and P5). They were more interested in fostering a community with other users than in the general content of the apps (P5). They identified 4 different needs for feeling connected to others: being able to share challenging content, seeing positive or motivational content, receiving support from others, and offering help to others.

Participants wanted to share their experiences and struggles with others who have similar experiences:

A safe space in which people who are experiencing the same feelings can talk to one another and just know that they’re not alone.

Participant not identifiable from the transcript

Participants valued having a space on the web to talk about things they would not be able to speak about with other people:

I feel like it’s for people that don’t have someone to talk to so they come on this app to like, I guess vent about their lives.

P5

P1 felt it was useful for them to receive support from a peer who was going through similar challenges than through generic content in the app:

Links to people who could help you out... who know what they’re doing...

P1

The participants also thought that offering support to others can bring them satisfaction as well. One participant posted a positive message on a community forum in the hope that it might help someone else who needed support (P5).

The participants valued authentic connections. They were reluctant to connect with others whose identities they could not recognize. For example, participants could not recognize if some of the social profiles of other people on the app were actual people or some type of automated account not associated with a person (P4).

#### Tracking Progress

Participants valued seeing their growth through tracking features, such as mood tracking or saved journal entries (P2). P2 noted that they felt they could not personally assess their emotional growth, and the app allowed them to reflect on their progress:

When you don’t track your progress, you don’t realize how far you come. But when you can physically see it, it can either be a positive thing or a negative, but sometimes is usually helps you adjust your mindset.

P3

#### Quick Engagement With Content

Some participants found it important to get value out of the apps quickly through short periods of engagement and lightweight interactions (P1 and P4). P4 mentioned that they valued audio content when they wanted to relax because they were able to get the support they needed to unwind quickly, or to complete an activity quickly, such as recognizing anxiety (P4). They did not like engaging with conversational apps because it took too much time to arrive at a helpful solution. They found it valuable when the apps were interactive; for example, conversational interfaces that felt like talking to a therapist (P4) or to another person (P3).

#### Ease of Use

Participants valued simplicity in apps: “simple and easy to use...you just know where to go, almost like you’re walking in your house” (P2) reflected in simple layouts, esthetics, and functionality. Participants wanted content to be easy to navigate and for them to quickly know how to use the app, “you just know...where to find everything” (P2). Participants thought that too many features were overwhelming:

There’s a lot of different features that pop up in the app...might feel overwhelmed.

P4

When apps contained too many features, it can make it difficult to find the desired content or even the content the user previously visited, which led to frustration:

Everything was all over the place in that app. It was hard to find a topic that I had seen earlier…couldn’t find it anymore.

P5

The participants felt meditation apps did this particularly well:

Especially meditation. And I feel like they’re simple enough…someone wouldn’t feel like they’re going out of their way.

P4

#### Match App Content With the User’s Context of Use

The participants “were more inclined to actually use the app” if the content was relevant to the need they had at the moment. In 1 instance, a participant used their app to wind down because the app had categories relevant to their intended use, to wind down at the end of the day. Because the user’s interests aligned with the app, they were able to engage with the app more meaningfully:

I was seeing an article about difficulty falling asleep sometimes. So, I was really interested in the article about sleep.

P4

### Reliability and Accessibility of Content

#### Accessibility of Apps and of Free Content

The participants identified certain aspects that made it hard for them to engage with the app. For example, 1 app included a community feature where people posted in foreign languages. P5 was concerned that some people might be expressing a need for help through their posts but that they might not be able to provide help because they do not understand the language.

The participants valued the availability of free content in the app and found frequent requests for upgrading to a paid version disruptive:

It was really pushing…need to buy…the premium….every time I go on the app.

P2

The participants preferred using apps that were richer in free content:

This app was definitely way better. I feel like there was more content that was free and available to public use.

P3 and P5

The trial version of apps also made participants reluctant about the benefits of the app after the trial period.

#### Reliability of Content

Some participants found it important to know that the content of the apps was validated by professionals and that it was not biased (P1). They wanted to ensure that people could connect with professions if they wish to:

I kind of feel like at that stage, it should bring up links to professionals, because we’re dealing with mental health problems here.

P1

#### Between-Session Activities

Once we began the study, the number of student responses to between-session activities over Discord was disproportionately low, yielding only 2 user responses. During this time, we first posted messages on Discord using an account that presented as a bot sending updates. This did not receive responses, so we changed our updates to be posted by one of our undergraduate researchers. We also asked about Discord engagement during our focus group session and encouraged participants to respond to prompts. This prompted responses from another participant. However, the responses were consistent and overlapped with what we were learning in the focus groups. Therefore, after the second week, we discontinued the between-session activities because engagement was low, and we were not receiving additional information in the feedback sessions. We also thought it would be more beneficial to keep participant feedback confined to focus groups where it could be discussed more in depth.

#### Follow-up Survey

In the follow-up survey, we identified 2 themes related to overall feedback on the apps. *Organization* of the app was identified as an important aspect across all app categories (CBT, mindfulness, and miscellaneous). The participants reported that they preferred apps with a simple interface such that they were “to the point,” organized, and easy to navigate. They disliked the presence of too many tabs, features, or steps to work through to get to the support they wanted. *Payment* was the second theme. In general, the participants reported that they disliked when most features were only available in a premium, paid version of the app, and they also disliked seeing promotions of paid content. This aligned with comments made throughout the sessions that participants thought that these types of apps should be free or indicated that they would be unlikely to download an app that required payment when free versions exist.

On average, the participants reported that the apps had a small positive impact on their mental well-being across all apps. No one indicated that the apps had a negative impact, although “no impact” was reported for 1 CBT app, 2 mindfulness apps, and 1 miscellaneous app. Miscellaneous apps were ranked as the most impactful, followed by CBT apps and then mindfulness apps.

When asked how often they used their app during the week they were assigned to use it, the participants were most likely to report using the app for “a few days of the week.” Participants were slightly more likely to continue using mindfulness apps after the study compared with CBT or miscellaneous apps. Of the 5 participants, 2 (40%) reported that they continued to use their CBT and miscellaneous apps after the week they were asked to use it, and 3 (60%) participants reported continuing to use their mindfulness app after they were asked to do so. The average star ratings assigned to the app categories by the participants were 3 for CBT apps and 3.6 for both mindfulness and miscellaneous apps out of a total of 5 stars.

## Discussion

### Principal Findings

In this study, we developed and refined a process for obtaining feedback on various MHapps. Our resultant series of focus groups allowed us to gain initial insights about Latinx youth’s preferences for and within our selected set of MHapps and their views on the benefits and use of the apps, albeit from a small sample of participants. As we gained experience with our procedure, we made a few changes; for example, removing the between-session activities and adding a final session to get impressions across all the MHapps. Despite refining the methods over the course of the 5 focus groups, we still identified some consistent preliminary themes across these sessions that provide some impressions of Latinx youth’s views on MHapps.

Latinx youth wanted apps that were youth centric in terms of content and functionality. However, youth-centric content meant not just making age-appropriate content in terms of language, visuals, or examples, but it also meant making the interactions brief and fun. Our participants also emphasized the importance of MHapps being able to transcend their digital components and facilitate meaningful offline interactions. Our follow-up survey also showed that ratings of MHapps might differ based on the types of questions asked. Although the CBT apps had lower star ratings than the other apps (3.0 vs 3.6), they were rated as being more impactful than the mindfulness apps. We discuss our findings in subsequent sections, both insights gained from what we learned from the participants and reflections on our methods.

The youth wanted MHapps that were reflective of youth problems, interests, and technology use. Many existing MHapps, even those that claim to be focused on the youth, often contain minimal surface-level tailoring, such as changing examples or esthetics [53]. Other approaches include using techniques such as gamification that might appeal to some youth. On the other hand, others might find that games trivialize mental health issues and would appreciate a more serious approach [33]. Indeed, another study that used a workshop approach to ideate multiple ideas for well-being technologies with the youth identified interests in diverse engagement strategies and types of technologies [32]. Thus, it is worth noting that “youth” is not a homogenous population, and differences exist among different subpopulations (eg, specific age ranges, gender or gender identity, race, or ethnic background).

Our study focused on the subpopulation of Latinx youth. Although we cannot comment on which themes identified were specific to Latinx youth, as we did not have a comparison group, it is worth reflecting on some things noted. Our participants noted the importance of social connectedness through MHapps. The importance of social connection has been noted in other mobile health studies comparing Spanish and English speakers, finding that Spanish speakers emphasized feelings of social support, whereas English speakers emphasized introspection and self-awareness [54]. It is hard to separate how much of these findings are related to youth generally or Latinx youth specifically, and further work could replicate these methods across different subgroups to help lead to more specific and definitive design recommendations.

Youth also go through different developmental processes. Therefore, adopting a developmental science lens might introduce affordances for MHapps and other youth-focused technologies [55]. One proposed solution to better reflect youth preferences in MHapps aimed at youth is to include youth and various youth stakeholders in the design process [56]. Indeed, various co-design methods (eg, questionnaires, interviews, focus groups, interactive workshops, and meetings) might be helpful to solicit input from youth and can be selected based on the stage of development and type of information needed [34]. We used some co-design methods in this project to solicit information regarding the developed MHapps, but of course, these methods should precede fully developed products.

It is also worth reflecting on considerations for evaluation methods for MHapps. Several evaluation frameworks or systems exist, such as the American Psychiatric Association framework [57], One Mind PsyberGuide, and Framework to Assist Stakeholders in Technology Evaluation for Recovery to Mental Health and Wellness [58]. Although these frameworks are intended to assist decision-making by diverse stakeholders, including consumers, providers, advocacy organizations, payers, and health systems, they often require specialized knowledge. This results in evaluations being conducted by individuals with expertise to apply such evaluation frameworks. Therefore, although consumer input might have been solicited in the development of these frameworks [48,59], methods for incorporating bottom-up input from consumers, especially consumers from diverse backgrounds, are needed. Some measures are relevant for consumers, such as the user version of the Mobile App Rating Scale [60] or the System Usability Scale [61], but these are mostly questions, rather than processes, for soliciting feedback. Our approach had both processes and questions, and many of the questions we used for our follow-up survey were derived from these measures [48,60].

We also found discrepancies between the youth feedback depending on how questions were asked. For example, we found differences between apps that were viewed as most liked (ie, mindfulness), using a 5-star rating scale, and those seen as more impactful (ie, CBT and miscellaneous). Given the vast amount of information available through app stores, many researchers have used ratings to understand the user perspectives of MHapps [36,37]. However, this information might obscure more nuanced perspectives on apps. The specific discrepancies identified in our study, for example, for mindfulness apps and CBT, might reflect how this content is delivered to youth. It is worth noting that we did not collect formal outcome measures regarding the impact of these apps; therefore, our findings are entirely based on the youth’s subjective perspective. Research on traditional mental health services has found that youth preferences do not always align with better outcomes [62]. As such, it is possible that these apps might have meaningful impacts on wellness and clinical targets or that satisfaction and clinical outcomes are not aligned, but additional research would be needed to verify this. Consistent with our previous points about the importance of separating app discovery (identifying apps), activation (initiating use), and engagement (repeated and sustained use), it is possible that elements that contribute to satisfaction, such as esthetics and learnability, might lead to people starting with an MHapp but that clinical effectiveness (ie, benefiting) might lead to sustained use. Other work has identified that people do discuss different factors related to barriers and facilitators to use across early initiation and long-term use [63], and studies that further explore the contributions of satisfaction and benefits could be useful here. For example, people who experience early symptom change might be more likely to stop using an MHapp (ie, “happy abandonment”) but might be more likely to return to it if symptoms reoccur or to recommend it to others struggling with similar issues.

### Reflections on the Apps That the Youth Designed in the Last Session

In our final focus group session, we had the participants design their own apps using a web-based collaboration platform. Participants received a series of prompts that covered different common features they identified (ie, symptom tracking, distraction tools, games, information, and links to resources). The participants consistently identified simplicity as a major design feature, but other common features included information, meditation or mindfulness tools, and links to other resources. This is extremely similar to popular features identified by unemployed individuals and essential workers for MHapps intended to support distress during the COVID-19 pandemic [64]. A few of our participants also noted distraction tools, which were also identified in the study [64], especially among unemployed workers. However, these interests are somewhat inconsistent with common features within MHapps, with the finding that the most common features both alone and in combination are journaling and mood tracking [65]. In fact, MHapps containing only journaling and mood tracking combined accounted for 16.5% of the reviewed 278 MHapps [65]. Many MHapps tend to be complex and multifeatured; however, our participants came up with suggestions that were simple and targeted. The IntelliCare app suite was designed to include simple, single-featured apps [66], and an analysis of users of this suite found that participants tended to use focused subsets of the various apps available to them [67]. Especially for the youth, who tend to be tech-savvy and technologically engaged, if a given MHapp does not meet their needs or is overly complex, they might use other technologies, perhaps not specifically designed for mental health, to meet their needs. Efforts should be made to better understand how youth use MHapps as part of their digital ecologies, and what needs and opportunities are lacking from how they are currently using technology to support their mental health and well-being.

### Lessons on Methodology to Engage With Youth

#### Recruitment and Community Partnership

We engaged with youth who had already participated in an educational community. Our research group had also engaged with the community before. These youth were receptive to participating in the research, which might be because of the norms of the organization and partnership with the research team. They were also insightful and engaged in providing feedback, which might not have been true for youth recruited through other means. Nevertheless, working with youth from this organization helped build trust and collaboration more quickly. It also provided opportunities to present our findings back to the leadership in the organization to consider how these lessons could shape other efforts to involve their youth in activities or consider opportunities for technology to support their wellness.

#### Familiarity of the Participant Group and Research Team

The participants knew each other before the focus groups started. However, the research team did not and was not aware of any of their relationships. This can create imbalances in the group because the research team is an outsider to the group. For future work, we recommend considering a group that has less familiarity so that everyone, including the research team, gets to know each other in a balanced way. Alternatively, our research team could have spent more time getting to know the youth and building a rapport before shifting to data collection. Another approach would be switching to remote activities only after a synchronous focus group where participants met each other [68]. Other work engaging children and adolescents in participatory design [69,70], especially using remote activities made necessary by the COVID-19 pandemic, has similarly demonstrated the need to build rapport with children and adolescents both at the beginning and repeatedly throughout extended design activities as we conducted.

#### Engagement With the Youth and Data Collection

We found it beneficial to conduct icebreaker activities as part of the focus group sessions because it got the participants started with sharing information about themselves with the rest of the participants and with the research team. We recommend keeping the icebreaker activities short so that they provide a warm-up to the activities but do not take a significant amount of time. One of the primary facilitators of the focus group was an undergraduate student (KC), which we found beneficial because this student was closer to the age of the participants than our other facilitators (EA, SMS, and MN) and could relate to them more in the discussion. We also found it useful to use informal language, including emojis, in communication with the youth. Although we initially attempted to send updates from an account that simulated a bot, we found that youth had slightly more updates when one of our undergraduate team members (Ashley McKinnon) messaged the Discord channel. We attribute this to participants relating more to a human being than to a generic bot.

#### Using a Social Network for Data Collection

We used the Discord channel to communicate with youth between sessions. This was beneficial because they were already using Discord for other activities, and the Discord channel we created for this study was part of the wider Discord of the community. However, the research team was not familiar with the norms of the community and how it was used by the group. Our interactions and questions did not result in responses from participants on the web. We recommend that in the future, researchers use a social platform that is familiar to the participants but is separate from their existing communities (eg, a separate Discord server, a separate Slack channel, or a private group on a social network). This allows researchers to define the norms of the community and participation. Similar to guidelines in prior work [69], researchers could design activities that encourage using the platform early on, or during focus groups, to familiarize participants with how they are expected to use the platform.

### Limitations

This work was limited by its formative nature; that is, we worked with a small sample of youth who were recruited through 1 specific community partnership. However, we would also like to note other limitations in this work that might be helpful to other researchers who conduct work in similar areas. As previously noted, the youth population is not homogenous. The participants we worked with were in the age range of 15 to 21 years, which captures mid- to late adolescence. Researchers should be mindful about what age ranges of youth populations they are most interested in working with and also appreciate that in this period of rapid development, a developmental science lens to understand the interplay of youth and technologies is necessary. We also worked with only youth who identified as Latinx, and our results may not be representative of the views of MHapps in other populations. We might also have gained different insights if we used different categories of MHapps. Our last category was a miscellaneous category, and we could have either replaced the categories we used (ie, CBT or mindfulness) or extended the number of focus group sessions to focus on different categories of apps, such as tracking or peer support apps. Not every aspect of our project went as planned, and we had to adjust methodologies along the way; for example, removing between-session activities. We expect that other projects likely change their initial plans and encourage other researchers to share these deviations as an opportunity to help others conducting similar work. Our team did not include members with a Latinx personal background. It is possible that participants would be less likely to connect and open up with our team members if they shared a Latinx personal background or that our analyses did not capture some elements of the Latinx experience because of the positionality of our research team. Finally, it is worth calling out some specific things that we did not hear from the participants we worked with. For example, recent work has called out several aspects related to the digital divide that might be barriers to use among marginalized groups, such as cost and internet and device access [71]. It is hard to disentangle whether these concerns are not present among the youth generally, perhaps because they are the most tech-savvy, these youth specifically, perhaps because we recruited them from a nonprofit team focused on Science, Technology, Engineering, Arts, and Math, or related to our selection of apps, which focused on free products. Nevertheless, it is important to note that assumptions related to digital literacy and technology access are worth checking and rechecking as knowledge and access continue to evolve.

### Future Directions and Work

We identified a range of needs for personalization of MHapps for the needs of youth. The content of the app should have some amount of topical relevance to youth’s interests and situations. Future work is needed to understand the topics that are most relevant to the youth and in what way they should be presented. For example, how specific content can be so that it is relatable to many but still feels meaningful to those for whom it is intended. The youth valued playful and lightweight interactions as part of the apps. More work is needed to understand how to implement such designs as part of existing MHapps; for example, MHapp designs could include lightweight audio content, activities designed in a way that feels less serious, or pairing activities that are perceived as demanding with activities that make the user feel good at the moment.

Building on this work, we see various potential avenues for future studies. One would be to conduct similar workshops with more youth, additionally drawing in youth from different racial and ethnic backgrounds or for different age ranges. We also see multiple ways in which this work could go deeper—individual interviews with the youth, refined between-session activities, and additional quantitative measures to look at the impact of MHapps both while using the apps (ie, process measures) and at the end of participation (ie, outcome measures). We believe that interviews and between-session activities could help better understand youth’s early experiences with MHapps, or app activation as we described earlier, as well as continued and sustained use (ie, app engagement). Additional quantitative measures could help better understand the impact of MHapps on youth. Finally, although we noted that app discovery, activation, and engagement were all challenges, given that we provided a select list of MHapps for participants, we did not learn much about discovery beyond how they navigated the choices we gave them. Future work could also work with the youth who elect to use MHapps on their own to better understand why and how they use them. It is likely that not all mental health and wellness needs that the youth are attempting to address with technologies are being approached using MHapps. The youth might be using other technologies, such as social media and games, and further work could help better understand how the youth approach those technologies in addition to MHapps.

We conducted a co-design activity with the participants in the last focus group. Future work could use structured design activities with the youth to understand specific preferences related to MHapps, that is, the types of functionalities, content, and visual look and feel of MHapps as well as that interactions align with their technology use practices. Our work provides some high-level insights—it should be youth centric, simple, and focused; translating these insights into design guidelines and app features would be a useful next step.

### Conclusions

Given the growing number of MHapps and youth’s tendency to use these resources, understanding their perspectives on MHapps can help guide the development, evaluation, and implementation of these tools for the youth. Our study developed and used a multisession focus group design to introduce MHapps to youth participants, elicit feedback on those apps, and learn more about what youth might want in MHapps. This work adds to a growing body of research focused on understanding youth’s needs and interests with regard to mental health technologies while also attempting to identify affordances that such technologies might offer, especially through a developmental science lens. We highlighted emerging themes, such as the need for simple and tailored content and the intersection of MHapps with youth’s offline lives. Overall, the participants we worked with expressed enthusiasm for this space, but more work needs to support building MHapps that are effective for the youth and determining how best to make these tools available to youth and integrate them into their lives.

## Abbreviations

ARC: asynchronous remote community
CBT: cognitive behavioral therapy
MHapp: mental health app
